# Comparison of intrinsic exercise capacity and response to acute exercise in ICR (Institute of Cancer Research) mice derived from three different lineages

**DOI:** 10.1186/s42826-021-00094-0

**Published:** 2021-08-04

**Authors:** Dong-Joo Hwang, Ki-Chun Kwon, Dong-Hun Choi, Hyun-Keun Song, Kil-Soo Kim, Young-Suk Jung, Dae-Youn Hwang, Joon-Yong Cho

**Affiliations:** 1 Exercise Biochemistry Laboratory, Korea National Sport University, Yangjae-daero, Songpa-gu, Seoul, Republic of Korea; 2 Department of Microbiology and Immunology, INJE University College of Medicine, Inje-ro, Gimhae-si, Gyeongsangnam-do Republic of Korea; 3 College of Veterinary Medicine, Kyungpook National University, Daehak-ro, Buk-gu, Daegu, Republic of Korea; 4 College of Pharmacy, Pusan National University, Busandaehak-ro 63beon-gil, Geumjeong-gu, Busan, Republic of Korea; 5 Department of Biomaterials Science, College of Natural Resources and Life Science/Life and Industry Convergence Research Institute, Pusan National University, Busandaehak-ro 63beon-gil, Geumjeong-gu, Busan, Republic of Korea

**Keywords:** Exercise capacity, ICR mouse, Korl:ICR, Mitochondrial coupling efficiency

## Abstract

**Background:**

As a laboratory animal resource, the ICR mouse is commonly used in a variety of research fields. However, information on differences in exercise-related characteristics in ICR mice derived from different lineages and the underlying mechanisms remains to be elucidated. In this study, we investigated the intrinsic exercise capacity and a magnitude of response to acute exercise, and sought to identify mechanisms contributing to difference in Korl:ICR (a novel ICR lineage recently established by the National Institute of Food and Drug Safety Evaluation, Korea) and two commercialized ICR lineages derived from different origins (viz., A:ICR mouse from Orient Bio Com, the United States, and B:ICR mouse from Japan SLC Inc., Japan).

**Results:**

Results showed that despite no significant difference in body weight and weight-proportioned tissue mass of heart and skeletal muscles among groups, the relatively low intrinsic exercise capacity and exaggerated response to acute exercise were identified in B:ICR comparted with Korl:ICR and A:ICR, as reflected by total work and lactate threshold (LT). Also, the mitochondrial efficiency expressed as the complex 1 and complex 1 + 2 respiratory control ratio (RCR) values for cardiac mitochondrial O_2_ consumption in B:ICR was significantly lower than that in Korl:ICR with higher level of state 2 respiration by glutamate/malate and UCP3 expression in cardiac muscle.

**Conclusions:**

Taken together, these results indicate that the intrinsic exercise capacity of ICR mouse varies according to lineages, suggesting the role of cardiac mitochondrial coupling efficiency as a possible mechanism that might contribute to differences in the intrinsic exercise capacity and magnitude of response to exercise.

## Background

Laboratory animals have been used as an indispensable tool in biomedical research contributing to our understanding of biological phenomena. Among the diverse lineages of rodents supplied by major vendors of laboratory animals, the ICR mouse, developed in 1945 at the Institute of Cancer Research (ICR), is commonly used in the research field of genetics, neurobiology, immunology, and pharmacology (drug efficacy and safety). This has led to the development of several lineages of ICR mouse worldwide for various research purposes [1–3].

Korl:ICR, a novel mouse lineage recently developed in the ICR strain background by the National Institute of Food and Drug Safety Evaluation (NIFDS) in Korea, has been rotation-mated for over 19 generations and is currently under genetic and phenotypic verification [4, 5]. Recently, our research groups found that, with the exception of certain phenotypic characteristics, the responses of Korl:ICR mice to diverse chemicals and physiological stimulations (e.g., gastric injury, constipation inducer, and anticancer drug treatment) are similar to those of two other lineages of different origins (the United States and Japan) supplied by commercial vendors [3, 5–7].

In the field of exercise science, several studies have been conducted to better understand the effects of exercise and its clinical application from a metabolic perspective using laboratory animals, including the ICR mouse [8, 9]. Related studies have demonstrated the positive effects of exercise, including metabolic, neuromuscular, and neurological improvement under sedentary and pathological conditions [10, 11], while emphasizing that differences in intrinsic exercise capacity can affect the applicability and extent of its effect [12–14]. Meanwhile, several studies on phenotypic characterization of rodents have reported both intra- and inter-strain differences in the exercise-related phenotypes and the response to exercise through different types of exercise test [15–17]. Moreover, it has been suggested that the influence of genetic and epigenetic (e.g., physiological factors) background ranging from central to peripheral organs of different mouse lineages can be the underlying causes and determinants of intrinsic exercise-related phenotypes [12, 14, 18]. However, information on the difference in intrinsic exercise capacity and response to exercise in rodents derived from different origins and its underlying mechanisms remains to be elucidated.

Therefore, the aim of the present study was to investigate the difference in intrinsic exercise capacity and magnitude of response to exercise in ICR mouse lineages (viz., Korl:ICR mouse from Korea, A:ICR mouse from the United States, and B:ICR mouse from Japan) derived from aforementioned different origins. A better understanding of the intrinsic exercise capacity and magnitude of response to acute exercise of mice derived from different lineages at the physiological level will provide novel information in the field of exercise science and ensure proper application of these ICR lineages as one of the useful laboratory animal resources.

## Results

### Intrinsic exercise capacity based on total work and lactate threshold in different ICR mouse lineages

**Fig. 1:**
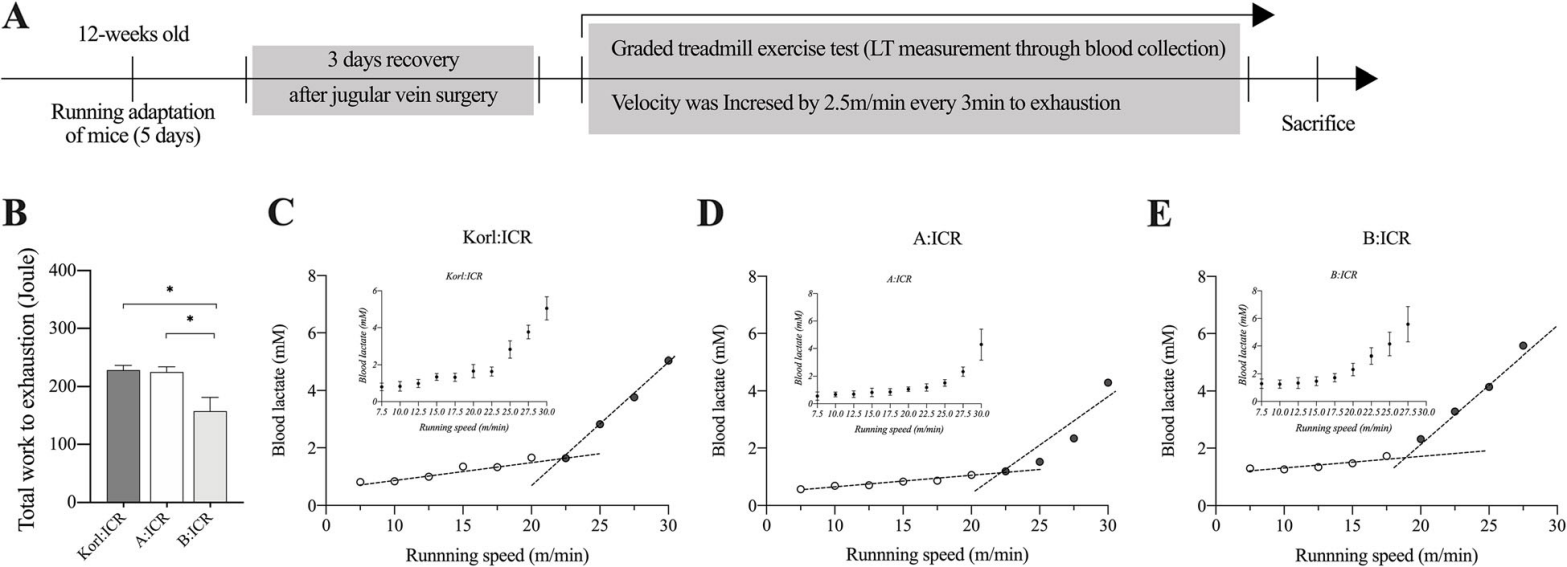
Identification of the total work and lactate threshold (LT) as direct and indirect indicators of exercise capacity. **A** Schematics of the experimental design and protocol. **B** TW to exhaustion through the graded treadmill running was calculated using a formula. **C**, **D**, and **E** The LT was determined by blood sampling via a jugular vein catheter during the graded treadmill exercise test. The values are presented as mean ± SEM; each group consisted of five mice. *P < .05

We measured the TW, and LT, as approximations of exercise capacity (Fig. 1A). A higher TW to exhaustion was observed in Korl:ICR and A:ICR mice (both 471 m in total) than those in B:ICR mice (Fig. 1B; total 325.5 m; TW, *F*_(2,12)_ = 6.734, *p* = .011). Consistent with the TW results, the LT measured in the blood sampled from the jugular vein was relatively lower in B:ICR mice than in Korl:ICR and A:ICR mice. For each ICR lineage, the LT was detected within a similar range of speed (approximately 25 m/min in Korl:ICR, 27.5 m/min in A:ICR, and 22.5 m/min in B:ICR; Fig. 1C, D and E).

### Changes in body weight and muscle mass after acute exercise

The body weight and mass of skeletal (soleus and EDL) and cardiac muscles of the three ICR lineages are shown in Table 1. There was no difference in the body weight of different ICR mouse lineages with varying exercise intensity (average: 40.79 g). Similarly, there was no difference among the lineages with respect to the ratio of muscle weight to total body weight.

**Table 1:** Comparison of physiological profile among different lineages of ICR mouse

	Control			Low intensity			Moderate intensity			High intensity		
	Korl:ICR	A:ICR	B:ICR	Korl:ICR	A:ICR	B:ICR	Korl:ICR	A:ICR	B:ICR	Korl:ICR	A:ICR	B:ICR
Body Weight (g)	41.6 ± 0.89	40.8 ± 1.16	40.3 ± 1.75	40.4 ± 1.14	42.0 ± 1.09	40.6 ± 1.30	41.4 ± 1.67	41.1 ± 0.98	39.7 ± 1.38	40.0 ± 1.58	41.5 ± 1.87	40.2 ± 1.49
Soleus muscle / BW (%)	0.05 ± 0.007	0.05 ± 0.01	0.04 ± 0.005	0.04 ± 0.007	0.05 ± 0.01	0.04 ± 0.007	0.05 ± 0.005	0.05 ± 0.004	0.05 ± 0.006	0.05 ± 0.009	0.04 ± 0.008	0.05 ± 0.01
EDL muscle / BW (%)	0.06 ± 0.004	0.08 ± 0.01	0.06 ± 0.009	0.07 ± 0.01	0.06 ± 0.008	0.06 ± 0.004	0.06 ± 0.006	0.07 ± 0.008	0.07 ± 0.005	0.07 ± 0.009	0.06 ± 0.004	0.07 ± 0.005
Heart / BW (%)	0.46 ± 0.02	0.45 ± 0.03	0.46 ± 0.03	0.51 ± 0.04	0.49 ± 0.03	0.49 ± 0.02	0.50 ± 0.05	0.54 ± 0.03	0.51 ± 0.01	0.49 ± 0.01	0.51 ± 0.045	0.50 ± 0.03

*BW* Body weight, *EDL* Extensor digitorum longus. The values are presented as mean ± standard deviation (SD); each group consisted of 5–6 mice

### Analysis of blood parameters associated with stress response and muscle damage immediately after acute exercise

**Fig. 2:**
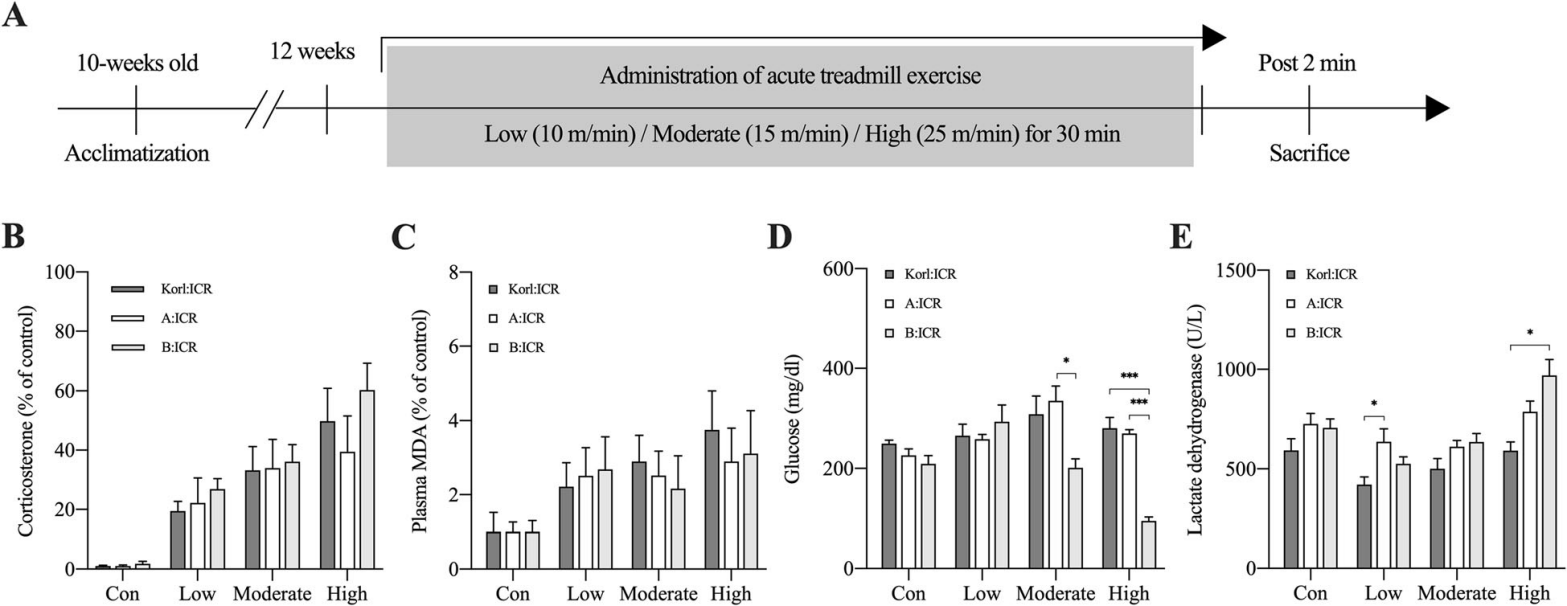
Comparative analysis of the magnitude of response to different acute exercise intensities (low, moderate, and high) among different lineages of ICR mice. **A** Schematics of the experimental design and protocol. **B** Changes in the blood corticosterone and **C** malondialdehyde levels. **D** Changes in the blood glucose, and **E** lactate dehydrogenase. The values are presented as mean ± SEM; each group consisted of 5–6 mice. *P < .05, **P < .01, and ***P < .001

At the end of the exercise session, there was no difference in the relative level of serum corticosterone and plasma MDA among the ICR mouse lineages, although these two variables showed a tendency to increase linearly with increase in exercise intensity (Fig. 2B and C).

To quantify the level of blood glucose and exercise-induced muscle damage immediately after acute exercise, we examined the level of blood glucose and activity of serum LDH as indices of muscle damage (Fig. 2D and E). The B:ICR mice showed a relatively rapid decline in the blood glucose level when subjected to moderate- and high-intensity exercise (Fig. 2D; moderate, *F*_(2,14)_ = 6.702, *p* = .009; high, *F*_(2,14)_ = 68.73, *p* < .001). Whereas, the activity of LDH in B:ICR mice subjected to high-intensity exercise was higher than that in Korl:ICR mice (Fig. 2E; high, *F*_(2,14)_ = 8.507, *p* = .004).

### Expression level of OXPHOS complex protein in cardiac muscle and cardiac mitochondrial respiration

**Fig. 3:**
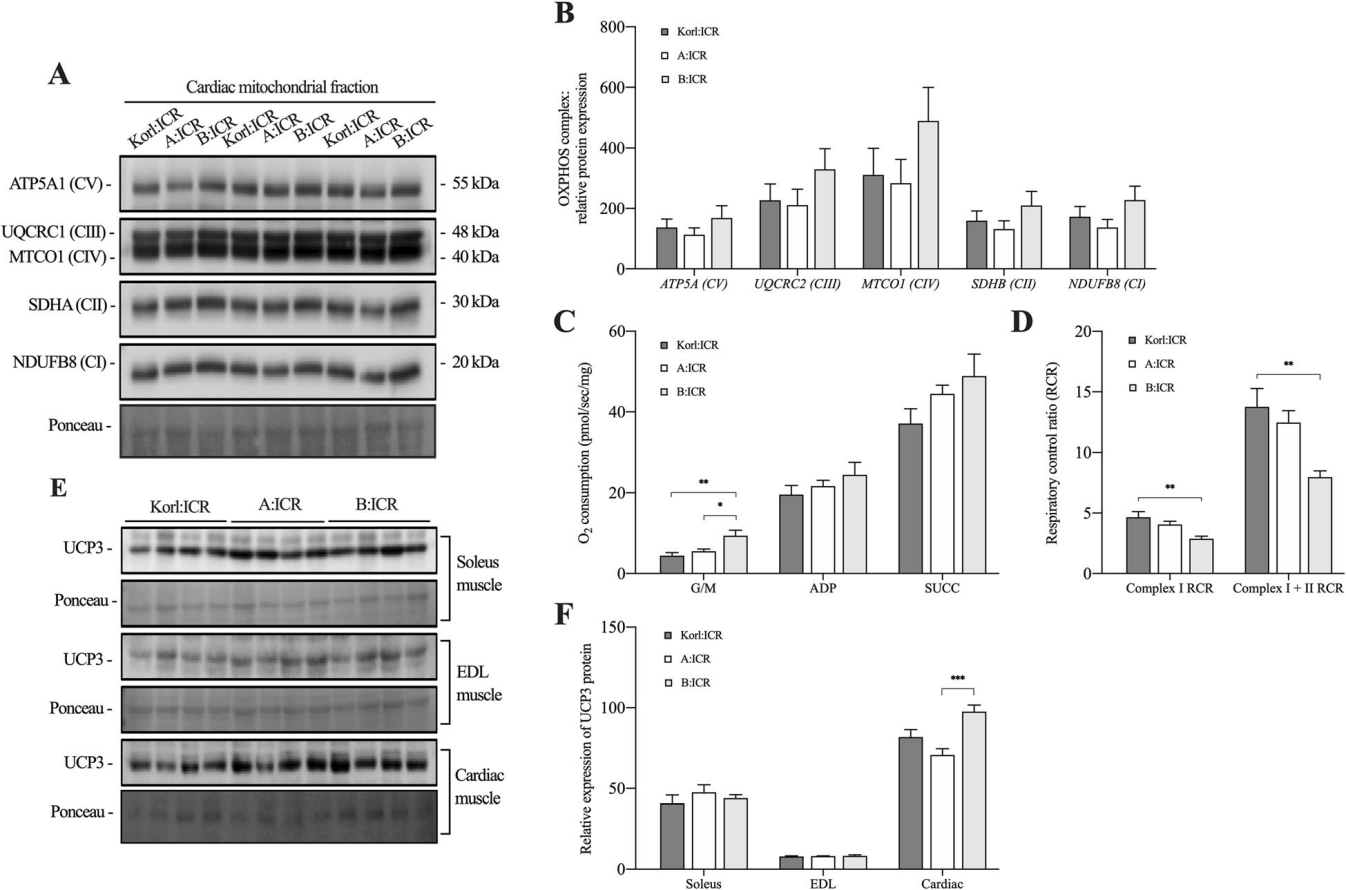
Comparative analysis of the cardiac mitochondrial oxidative phosphorylation (OXPHOS) complex, mitochondrial function, and uncoupling protein (UCP) 3 expression levels among the ICR lineages. **A** Representative western blots showing the protein levels of the OXPHOS complex. **B** The densitometric analysis of blots normalized for COX-IV in the cardiac mitochondrial fraction of different ICR lineages. **C** Oxygen consumption rate (OCR) in the permeabilized cardiac muscles of different ICR lineages was recorded using the Oroboros O2K instrument. **D** The respiratory control ratio (RCR) was calculated as the ratio of oxygen consumption at state 3 (complex 1 and complex 1 + 2) to that at state 2. **E** Representative western blots showing the protein levels of UCP3 in the soleus, extensor digitorum longus (EDL), and cardiac muscle. **F** The densitometric analysis of blots normalized for the density of Ponceau staining in different muscles. The values are presented as mean ± SEM; each group consisted of 5–6 mice. *P < .05, **P < .01, and ***P < .001

We performed quantitative and functional analyses of OXPHOS complex constitutive proteins in the myocardial mitochondria of the three ICR lineages (Fig. 3A and B). Our results showed no difference among the lineages with respect to the relative expression level of OXPHOS complexes I to V. However, complex 1 state 2 respiration, as expressed by the substrate response (G/M), in B:ICR mice was higher than that in other ICR mice (Fig. 3C; *F*_(2,14)_ = 7.941, *p* = .005). Whereas, complex 1 RCR and complex 1 + 2 RCR for cardiac mitochondrial O_2_ consumption in B:ICR mice was lower than that in Korl:ICR mice (Fig. 3D; complex 1 RCR, *F*_(2,14)_ = 6.704, *p* = .009; complex 1 + 2 RCR, *F*_(2,14)_ = 6.678, *p* = .009).

### Expression level of uncoupling protein 3

We examined the expression level of UCP3 protein, which is known to affect mitochondrial coupling efficiency in the cardiac and skeletal muscles (soleus and EDL) of ICR mouse lineages (Fig. 3E and F). As expected based on our observations of mitochondrial O_2_ consumption, the level of UCP3 protein in the cardiac muscle of B:ICR mice was higher than that in the cardiac muscle of other lineages (Fig. 3F; *F*_(2,15)_ = 10.296, *p* = .002). However, we were unable to detect any differences among the ICR mouse lineages with respect to the level of UCP3 in the soleus and EDL (Fig. 3F).

### Determination of the effects of cardiac mitochondrial proton leakage on resting metabolic rate during the mature adult phase

**Fig. 4:**
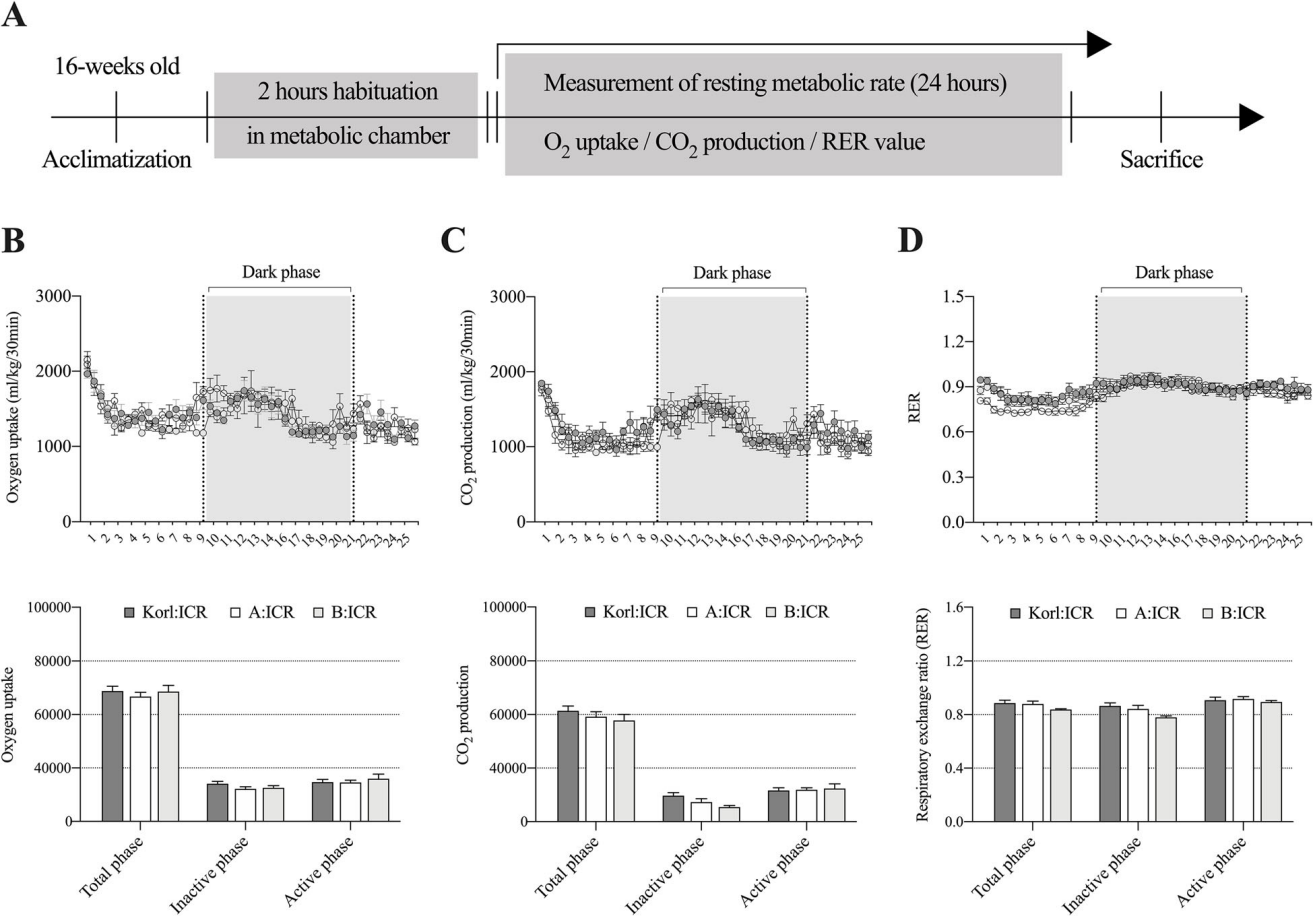
Comparative analysis of the metabolic phenotypes of different ICR lineages during a 24-h period at resting metabolic rate (RMR). **A** Schematics of the experimental design and protocol. **B** Changes in oxygen uptake and total oxygen uptake during different phases (total, inactive, and active). **C** Changes in CO_2_ production and total CO_2_ production during different phases. **D** Changes in the respiratory exchange ratio (RER) and average RER during different phases. The shaded part of the line graph represents the dark period of the day cycle. The values are presented as mean ± SEM; each group consisted of four mice

We further examined whether functional differences in cardiac mitochondria affect the metabolic rate under 24-h resting conditions (Fig. 4A - D). We found no differences in the RMR (total period, inactive period, and active period) of the ICR lineages expressed by O_2_ uptake, CO_2_ production, and the respiratory exchange rate (RER).

## Discussion

To date, several studies have shown that there are certain differences in the physiological and metabolic responses among mouse strains to exercise stimulus [14, 19]. However, the metabolic changes related to physical exercise among different mouse lineages are poorly understood. In the present study, we investigated the intrinsic exercise capacity and response to acute exercise in ICR mouse lineages derived from three different sources, including the newly developed Korl:ICR mouse lineage, to determine mouse lineage-specific differences and elucidate the possible underlying mechanisms.

Interestingly, in terms of intrinsic exercise capacity based on the TW to exhaustion and the level of LT, our results showed that Korl:ICR and A:ICR mice have a relatively higher exercise capacity and exercise endurance than B:ICR mice do. Our results imply that the exercise-related physiological characteristics of B:ICR mice have a partially exaggerated reactivity compared with those of Korl:ICR and A:ICR mice. Several studies that have investigated differences in the exercise capacity of rodents have mainly discussed the differences among strains with a wide range of genetic diversity. Avila et al. [12] reported that the strain with the highest exercise capacity ran twice as long as the lowest and Massett & Berk [14] reported the difference in exercise-related phenotypes of rodents was also observed in hybrid mice. Thus, despite certain uncontrollable or unidentifiable factors related to the experimental environment or testing protocol used, these results exhibited a similar tendency to those of previous studies, demonstrating the difference in intrinsic exercise capacity by the influence of genetic background.

To further validate our aforementioned results, we investigated the difference in the magnitude of response to acute exercise. Consistent with our recent observations and those of other studies involving the same ICR lineages [3, 5–7], the results revealed a higher level of LDH in B:ICR mice than in Korl:ICR mice, accompanied with a sharp decline in the blood glucose level coupled with an increase in exercise intensity. However, we did not observe significant mouse lineage-dependent differences with respect to biological properties (e.g., body weight and muscle tissue mass) and the level of corticosterone and MDA, although they showed a tendency to increase linearly with increase in exercise intensity. With respect to exercise-related physiological characteristics in the three lineages of ICR mice, B:ICR mice exhibited an exaggerated physiological responses in GLU and LDH to acute exercise. It has been suggested that hypoglycemic response and the elevation of LDH by prolonged and acute exercise are typically shown in human and rodents [20–23], which basically means that B:ICR mice might have had relatively lower metabolic efficiency despite the similar physiological profile. These findings indicate that the ICR lineages with different physiological characteristics by genetic background might have some differences in exercise-related phenotypes, such as lower exercise capacity observed in B:ICR mice, in terms of metabolic and hematological responses.

Recently, in addition to the direct influence of skeletal muscle function on exercise capacity, exercise endurance has been evaluated. Several studies have examined cardiac LV function based on the energy flux of myocardium considering oxygen delivery and fatigue resistance through fine-tuning of the skeletal muscle and myocardium function [24, 25], as well as the pathological exercise intolerance in patients with heart failure [24, 26, 27]. In the present study, to elucidate the underlying mechanism of difference in exercise capacity among ICR lineages based on the experimental verification of previous studies, we focused on cardiac function to identify factors that might influence the intrinsic exercise capacity and other exercise-related physiological variables mentioned above at the molecular level.

A comparison of enzyme expression level and the mitochondrial oxygen consumption rate of the OXPHOS complex in the cardiac LV muscle revealed that B:ICR mouse has a significantly higher complex 1 substrate (G/M) response (state 2) than that of the other two lineages. These responses ultimately lowered the RCR in B:ICR mice compared with that in Korl:ICR mice, indicating a strong coupling between respiration and phosphorylation [28, 29]. Although further studies are necessary to elucidate the physiological functions, the fact that complex 1 state 2 respiration is an index of proton leakage suggests that a relatively low coupling efficiency in B:ICR mice might have affected their intrinsic exercise capacity [29]. In accordance with the aforementioned results, the relatively higher expression of UCP3 in B:ICR mice, a protein that mediates ATP synthesis and energy expenditure through coupling in the cardiac LV muscle, is consistent with our results of cardiac function and the findings of previous studies, that is, metabolic disorders have a correlation between UCP3 and cardiac efficiency [30–32]. Additionally, the difference in UCP3-mediated cardiac efficiency [30], as an element affecting the RMR by regulating the coupling efficiency, did not affect the factors associated with the RMR, such as O_2_ uptake, CO_2_ production, and respiratory exchange ratio (RER: a value obtained from the VCO_2_/VO_2_ ratio), during the mature adult phase of mice [33]. Considering that UCP3-mediated cardiac efficiency is related to aging [34], the present study suggest that the metabolic efficiency of cardiac mitochondrial OXPHOS observed in B:ICR mice is estimated to be closely associated with exercise-specific response to young adult phase of ICR mouse lineage.

A limitation of our study was that we interpreted the experimental results based on the exercise physiological theories, lacking results of the cardiovascular system’s functional aspects and muscular system analysis directly related to the exercise-related phenotypes. Therefore, further studies should carry out comprehensive and logical verification of these results, which would provide valuable information in the field of exercise science and laboratory animals.

## Conclusions

In the present study, we demonstrated certain ICR lineage-specific differences in exercise-related physiological characteristics and its underlying mechanism that might possibly contribute to differences in the magnitude of response to exercise and exercise capacity. The results of the present study can be a starting point for ICR mouse lineage selection in the field of laboratory animal resource and exercise science. Additionally, given that the present and previous studies have indicated that the magnitude of overall responses of Korl:ICR mice to various treatments is similar to that of commercialized ICR lineages, Korl:ICR mice can be used as an alternative mouse model for various studies.

## Methods

### Animals

All applicable international, national, and/or institutional guidelines for the care and use of animals were followed. The procedures used for animal experimentation were approved by the Institutional Animal Care and Use Committee of Korea National Sport University (Approval Number: KNSU-IACUC-2017-10). Male ICR mouse lineages (10 weeks old) were obtained from three different sources considering the age range for assessing exercise-related phenotypes [12, 35]. Korl:ICR mice were provided by the Department of Laboratory Animal Resources at the National Institute of Food and Drug Safety Evaluation (NIFDS, Cheongju, Korea) and the other mice were purchased from different commercial vendors, namely, Orient Bio Com. (Gyeonggi-do, Korea) and Japan SLC Inc. (Shizuoka, Japan). All mice were acclimatized to pathogen-free laboratory conditions (12:12 h dark–light cycle, 22 ± 2 °C, and 50% relative humidity) for 2 weeks at the laboratory animal facility of Korea National Sport University. Food and water were provided ad libitum, according to the schedule for laboratory animals.

### Exercise protocol

The mice were made to perform acute exercise on an eight-lane motorized rodent treadmill (Dae-myung Scientific Co. Ltd., Korea). Prior to the treadmill test, the mice were habituated to the treadmill for two consecutive days at a speed of 8 m/min for 10 min/d at a gradient of 0°. After the habituation period, the mice were subjected to three different intensities of acute exercise, which were selected based on a previous study [36]: low intensity (10 m/min), moderate intensity (15 m/min), and high intensity (25 m/min) for 30 min (*n* = 5 per group).

### Calculation of total work

Total work (TW) to exhaustion through the graded treadmill running, as one of the parameters for evaluating the exercise capacity among the different ICR mouse lineages, was calculated using the following formula:

$$ \mathrm{Work}\ \left(\mathrm{J}\right)\kern0.5em =\mathrm{Body}\kern0.5em \mathrm{m}\mathrm{ass}\kern0.5em \left(\mathrm{kg}\right)\kern0.5em \times \kern0.5em \mathrm{Gravity}\kern0.5em \left(9.81\kern0.5em \mathrm{m}/{\mathrm{s}}^2\right)\kern0.5em \times \kern0.5em \mathrm{Vertical}\kern0.5em \mathrm{speed}\kern0.5em \left(\mathrm{m}/\mathrm{s}\kern0.5em \times \kern0.5em \mathrm{angle}\right)\times \kern0.5em \mathrm{Time}\left(\mathrm{s}\right) $$

### Measurement of lactate threshold

As an indirect evaluation of exercise capacity, we determined the blood lactate threshold (LT), an indicator of exercise intensity, via jugular vein catheterization using a method previously reported [36]. The LT was determined after maintaining the mice in a standard cage for 2 d after surgery. The treadmill exercise protocol for LT measurement was as follows. The initial treadmill velocity was 5 m/min, which was then increased at a rate of 2.5 m/min every 3 min until the point of exhaustion (a state in which a mouse was unable to keep pace with the treadmill velocity and remained at the back of the treadmill for over 30 s despite a gentle nudge) [37, 38]. Blood samples were collected via a syringe connected to the catheter 30 s before each increase in treadmill velocity and analyzed using an automated lactate analyzer (1500 Lactate Analyzer; YSI, USA). A statistical approach to evaluate the LT was established indirectly based on significant differences between the values at the time of increase in exercise intensity and during the previous step.

### Tissue preparation

Immediately after the acute exercise administration, a carbon dioxide (CO_2_) euthanasia method commonly used in small rodents was applied to experimental animals. In brief, mice were placed in an enclosed chamber and then gradually increased the concentration of CO_2_ to induce sleep, followed by hypoxia leading to euthanasia. After that, the soleus (a slow-twitch fiber), extensor digitorum longus (EDL; a fast-twitch fiber), and cardiac muscle (left ventricle; LV) were extracted, and their wet weight was measured using an electronic balance (Mettler Toledo, USA). The muscle samples (*n* = 5–6 per group) for western blotting were thoroughly washed with 0.1 M phosphate-buffered saline (PBS), rapidly frozen in liquid nitrogen, and then stored in a − 80 °C deep freezer (SANYO, Japan) until further use.

### Hematological analysis

The concentration of corticosterone and malondialdehyde (MDA, an indicator of lipid peroxidation) in the blood samples collected immediately after the acute exercise was determined using a commercial ELISA assay kit (CORT, ab108821; MDA, ab118970), following the manufacturer’s instructions. Briefly, to prepare the respective standard curves and determine the concentration of corticosterone and MDA, different concentrations of corticosterone and MDA standard solutions were added to the wells of 96-well microplates and the serum and plasma samples were dispensed in separate wells. The reaction in the 96-well microplate was run for approximately 20 min at room temperature ranging from 20 °C to 25 °C. After ceasing the reaction by adding the stop solution, the concentration of corticosterone and MDA in the plasma and serum samples was calculated from the standard curves by measuring the absorbance of the samples (at a wavelength of 450 nm for corticosterone and 532 nm for MDA) using a microplate reader (HIDEX Sense, Finland).

Furthermore, to determine the level of blood glucose (GLU) concentrations and activities of lactate dehydrogenase (LDH) as indicators of muscle damage, the blood samples were incubated at room temperature for approximately 1 h and centrifuged at 3000 rpm for 10 min (Beckman, USA). The extracted serum was transferred to an Eppendorf tube and analyzed using a HITACHI 7080 blood analyzer (HITACHI, Japan).

### Isolation of mitochondria

Mitochondrial fractions were obtained from the cardiac muscle tissues (left ventricle; LV) using a commercial Mitochondria Isolation Kit (NOVUS IMGENEX Corporation, San Diego, CA) following the manufacturer’s instructions. Following homogenization, the brain tissues were centrifuged at 3000 rpm for 10 min (4 °C), and the resulting supernatant was again centrifuged at 12,000 rpm for 30 min (4 °C). Thereafter, the supernatant (cytosolic fraction) was discarded, and the pellet was suspended with suspension buffer and centrifuged at 12,000 rpm for 10 min (4 °C). The pellet obtained was suspended with mitochondrial lysis buffer, allowed to stand for 30 min at 4 °C, and centrifuged at 12,000 rpm for 5 min (4 °C); the supernatant obtained was collected as the mitochondrial fraction.

### Mitochondrial respirometry

Mitochondrial oxygen consumption, an index of mitochondrial function, in cardiac muscle tissues (left ventricle; LV) was measured using an Oroboros Oxygraph-O2K device (Oroboros Instruments, Innsbruck, Austria). After calibration of the equipment, the following process was carried out. Approximately 10 mg wet weight of LV tissue was permeabilized using assay buffer Z (105 mM K-MES, 30 mM KCl, 10 mM KH_2_PO_4_, 4 mM MgCl_2_-H_6_O_2_, and 0.5 mg/mL BSA; pH 7.1). The permeabilized sample was subjected to respirometry analysis [28]. Oxygen uptake by mitochondria was measured under the following conditions: (I) glutamate + malate (G/M, complex I substrate, state 2), (II) ADP (state 3), and (III) succinate (complex II substrate). The mitochondrial oxygen consumption is expressed as pmol/s/mg (wet weight). The respiratory control ratio (RCR) was obtained by dividing the ADP-stimulated respiration by state 2.

### Western blotting

Tissues (soleus, EDL, and cardiac muscle) were homogenized in the RIPA lysis buffer (ELPIS Biotech, Korea) and the concentration of proteins was determined by Bradford method. The proteins (30 μg) were separated by sodium dodecyl sulfate polyacrylamide gel electrophoresis (8% or 12% separating gel), transferred on to polyvinylidene difluoride (PVDF) membranes (Millipore, Boston, MA), and blocked with 5% bovine serum albumin. The membranes were incubated with the primary antibodies overnight at 4 °C. As primary antibodies, we used a total oxidative phosphorylation (OXPHOS) complex cocktail (ab110413, diluted in 1:1000) and uncoupling protein 3 (UCP3; sc-31,385, diluted in 1:1000). The membranes were then washed with washing buffer (phosphate-buffered saline containing 0.1% Tween 20), and then incubated with horseradish peroxidase (HRP)-conjugated secondary antibody (Invitrogen; dilution, 1:5000). The level of protein expression was determined using the Luminate Forte Western HRP Substrate (Millipore, USA). The density of the protein bands developed was monitored using a ChemiDoc XRS system (Bio-Rad, Hercules, CA, USA).

### Measurement of metabolic phenotypes

The resting metabolic rate (RMR), an index of metabolic phenotype, was measured in mice at the age of 16 weeks (the mature adult phase of mice equivalent to human age of 20–30 years) [39]. The RMR was measured for 24 h in a metabolic chamber using an open-circuit system (WSMR-1400; Westron, Japan) as previously described [40]. To minimize stress, the mice were transferred to the chamber 2 h prior to the measurements. The flow rate within the chamber was set at 1.2 L/min, and one of the chambers was used to measure the ambient air for calibration purpose. A mass spectrometry-based gas analyzer (RL-600; Alco System, Japan) equipped with a switching system (ANI6-A-S; Alco System, Japan) was used to measure O_2_ uptake, CO_2_ production, and the respiratory exchange rate (RER).

### Statistical analyses

All graphs and tables were prepared using GraphPad Prism software (GraphPad Prism Inc., CA, USA) and the data were analyzed using SPSS version 22.0 (SPSS Inc., Chicago, IL, USA). Statistical significance was determined by the one-way analysis of variance (ANOVA) followed by the Bonferroni post hoc test for multiple comparisons. All values are expressed as mean ± standard error (SEM) and statistical significance was tested at *p* < 0.05.

## Data Availability

Available.

## Abbreviations

ICR: Institute of Cancer Research
TW: Total work
LT: Lactate threshold
OXPHOS: Oxidative phosphorylation
RCR: Respiratory control ratio
UCP3: Uncoupling protein 3
CORT: Corticosterone
MDA: Malondialdehyde
GLU: Glucose
LDH: Lactate dehydrogenase
